# Case report: Immune-mediated meibomian gland dysfunction following pembrolizumab therapy for advanced urothelial carcinoma

**DOI:** 10.3389/fonc.2022.1000023

**Published:** 2022-10-06

**Authors:** Charles B. Nguyen, Christopher T. Su, Meredith Morgan, Ajjai S. Alva

**Affiliations:** University of Michigan Rogel Cancer Center, Ann Arbor, MI, United States

**Keywords:** immunotherapy, immune-related adverse events, ocular toxicity, meibomian gland dysfunction, immunotherapy adverse reactions

## Abstract

Ocular immune-related adverse events are a relatively rare complication of immune checkpoint inhibitors. Common ocular toxicities range from dry eyes to inflammatory uveitis and ocular myasthenia gravis. Here, we present the case of a 55-year-old woman with recurrent urothelial carcinoma of the ureter after initially being managed with neoadjuvant cisplatin-based chemotherapy and surgical resection. She was treated with pembrolizumab which was complicated by immune-mediated pneumonitis after the eighth cycle, which was managed with a prolonged steroid course. The patient also developed red eyes along with recurrent styes. Eye examination revealed decreased tear breakup time, expression of thick and turbid meibum, and meibomian gland atrophy on infrared meibography. The patient was diagnosed with suspected immune-mediated meibomian gland dysfunction (MGD) as a result of pembrolizumab, a previously unreported complication of immunotherapy. The goal of MGD therapy is to stabilize the tear film and minimize evaporation with lipid-based lubricants and other conservative treatments.

## Introduction

Recent cancer treatments have been dramatically transformed with the development of immunotherapy. Immune checkpoint inhibitors (ICIs) are monoclonal antibodies targeting regulatory proteins involved in T cell activation such as PD-1, PD-L1, and CTLA-4 thereby enhancing cytolytic activity against cancer cells. Despite positive responses with immunotherapy, ICIs are associated with a variety of immune-related adverse events (irAEs) that involve multiple organ systems including lung, gastrointestinal, skin, and endocrine system. Ocular and ophthalmic irAEs are exceptionally rare, occurring in approximately 1% of patients (1). In an analysis of the Food and Drug Administration’s (FDA) adverse events database, nivolumab was associated with the most number of ocular irAEs and accounted for over a half of cases (2). Combination of anti-CTLA-4 and anti-PD-1 antibodies was also associated with a higher proportion of ocular irAEs compared to monotherapy (3).

**Figure 1:**
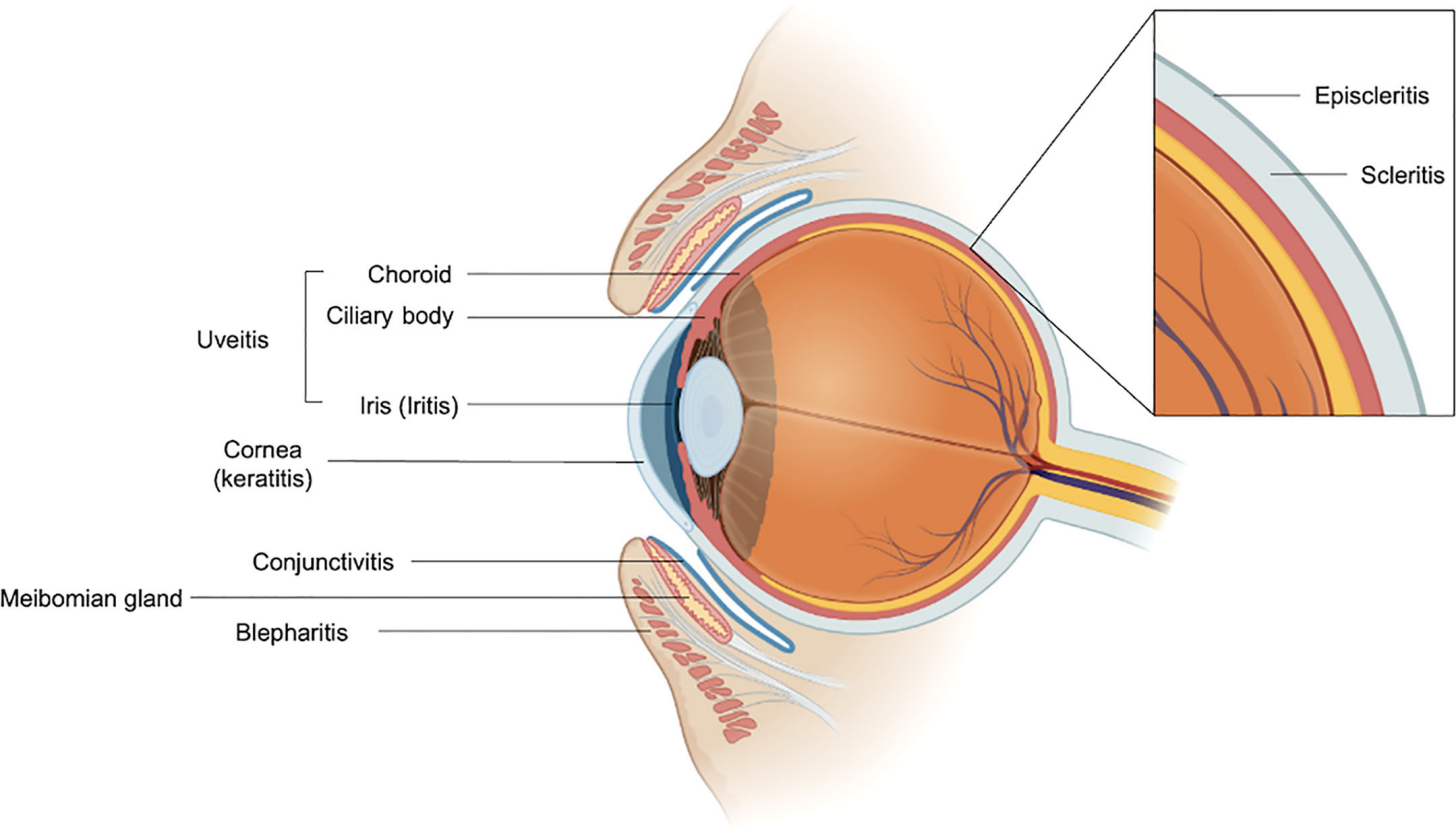
Anatomic organization of reported ocular immune-mediated adverse events. The meibomian gland is labeled for reference. Figure was generated using BioRender.com.

Most ocular irAEs can be classified based on the anatomic compartment affected (**Figure 1**). Common ocular irAEs that have been reported are dry eyes (1-24% among reported ocular irAEs), inflammatory uveitis (1-15% among reported ocular irAEs), and ocular myasthenia gravis (n = 19 cases reported) (1, 2). Other reported ocular irAEs include blepharitis, episcleritis, and iritis (all reported in < 1% of patients receiving anti-CTLA-4 antibody therapy) as well as keratitis (n = 3 cases documented) (1, 4–6). Patients may commonly present with symptoms such as dry eyes, blurry vision, eyelid swelling, and conjunctivitis (7, 8). Immune-mediated uveitis can present with photophobia, visual changes, eye pain, and floaters (1). Evaluation by an ophthalmologist should be completed when any ocular symptoms develop. In most cases, mild ocular irAEs can be simply managed with conservative therapies such as artificial lubricants (8). For grade 1 uveitis and episcleritis, defined as mild eye symptoms, ICI therapy can be continued in conjunction with supportive care (9). Higher grades of uveitis such as posterior or pan-uveitis (grade 3) or 20/200 visual acuity (grade 4) are managed by holding ICI therapy and initiating ophthalmic or systemic steroids (9). Similarly, grade 2 to 4 episcleritis (characterized by degree of visual acuity) can also be managed with holding immunotherapy and using steroids (9).

Meibomian gland dysfunction (MGD) is a common cause of dry eyes that occurs due to ductal gland obstruction and has been previously reported in patients receiving systemic chemotherapy (10). However, MGD has not been reported in patients receiving checkpoint inhibitor immunotherapy. Here, we report a unique case of suspected immune-mediated meibomian gland dysfunction in a patient who received pembrolizumab therapy for recurrent urothelial carcinoma.

## Case presentation

A 55-year-old woman with no significant medical history initially presented with several months of right-sided flank pain. A computed tomography (CT) of the abdomen and pelvis revealed severe right-sided hydroureteronephrosis and right cortical atrophy of the right kidney. A CT urogram demonstrated a soft tissue mass involving the distal right ureter near the ureterovesical junction as well as enlarged right external iliac lymph nodes. She underwent cystoscopy which identified tumor arising from the right ureteral orifice. A transurethral resection of bladder tumor (TURBT) was performed which revealed invasive high-grade, poorly differentiated papillary urothelial carcinoma that extended into the lamina propria but did not invade into the muscularis propria. A biopsy of the right external iliac lymph node was positive for malignancy. The patient was thus initially diagnosed with stage IIIA disease (pT1N1M0) and started on neoadjuvant dose dense methotrexate, vinblastine, doxorubicin, and cisplatin (ddMVAC). She eventually underwent a radical nephroureterectomy, partial cystectomy, and pelvic lymph node dissection. At the time of surgery, she was found to have microscopic foci of malignancy with negative surgical margins and negative lymph nodes in the nine total lymph nodes examined. Adjuvant therapy was not indicated given the pathologic findings of pTaN0Mx, and the patient proceeded with surveillance.

Four months after surgery, she was found to have a new 3.1 cm right inguinal lymph node and 1.3 cm anterior aortocaval lymph node on re-staging CT imaging. Biopsy of the right inguinal lymph node was positive for recurrent urothelial carcinoma. There was concern for platinum resistance given her prior neoadjuvant ddMVAC therapy. The patient was therefore started on intravenous pembrolizumab 200 mg every 3 weeks instead of a cisplatin-based regimen. Re-staging CT imaging after approximately three months of pembrolizumab indicated decreasing size of the inguinal and pelvic lymph nodes. After five months of pembrolizumab (eight cycles), repeat imaging indicated no evidence of disease. However, CT chest imaging at this time revealed interval development of bilateral ground glass opacities in the lungs. At this time, the patient did not report any respiratory symptoms such as shortness of breath, dyspnea on exertion, or cough. However, pembrolizumab was held given the concern for immune-mediated pneumonitis. She eventually developed acute-onset shortness of breath and fever for which she presented to the hospital where she was found to have progressive bilateral ground glass opacities involving all of the lobes. Infectious workup including *Pneumocystis jirovecii* was negative. She was then started on a prolonged steroid taper for grade 3 immune-mediated pneumonitis. One month after starting steroids, she had improvement in respiratory symptoms. An interval CT chest at this time demonstrated improvement in the bilateral opacities. The patient completed her steroids over two months with complete resolution of her pulmonary symptoms.

**Figure 2:**
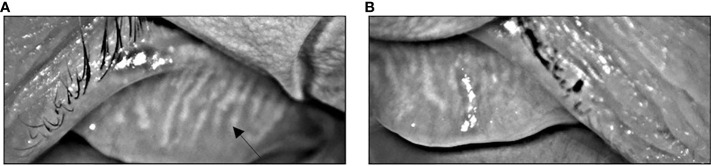
Infrared meibography showing atrophy and truncation (arrow) of the meibomian glands of the left **(A)** and right **(B)** eyes characteristics of MGD.

Approximately 4 weeks later (3 months after her last dose of pembrolizumab), she developed several recurrent episodes of red eyes, styes, and eyelid tenderness involving both eyes. She had pre-existing myopia and age-related presbyopia but did not have any changes in visual acuity. The patient did not have any blurry vision or ocular pain. Given that her symptoms persisted despite conservative measures, she was evaluated by an optometrist. Visual acuity examination was stable. Tear breakup time (TBUT) was low suggesting tear film instability. The patient was also noted to have thin tear meniscus in both eyes. Digital expression of the meibomian glands revealed thick, turbid meibum. Infrared meibography revealed bilateral grade 2 meibomian gland atrophy (**Figure 2**). Taken together, she was diagnosed with meibomian gland dysfunction (MGD). Management was started with warm compresses, lid massages, oil-based eye drops, as well as dietary omega-3 fatty acid supplementation. With these measures, the patient had significant improvement in her eye symptoms. Pembrolizumab was not continued after eight cycles given that her interval staging scans did not show any evidence of cancer or recurrence and since she had experienced severe pulmonary toxicity. At a follow-up two years after completing pembrolizumab, the patient was doing well clinically without evidence of cancer on repeat imaging. She continued to have mild eye symptoms and persistent meibomian gland atrophy on meibography. However, her symptoms were well-managed with continued MGD therapies.

## Discussion

Meibomian gland dysfunction (MGD), a common cause of dry eyes, is characterized by the International Workshop on MGD (IWMGD) as having “terminal duct obstruction and/or qualitative/quantitative changes in the glandular secretion.” (11) Located in the tarsal plates of the eyelids, meibomian glands function to produce and excrete lipids and proteins into the eye’s tear film which stabilizes and reduces evaporation of the tear film (11, 12). Several risk factors for the development of MGD have been identified. MGD has been associated with Asian populations, advanced age, contact lens use, *Demodex folliculorum* mite infestation, and medications such as anti-androgen agents and systemic 13-cis-retinoid acid (11, 13). MGD can also occur due to underlying eye conditions such as ocular rosacea (14). Administration of chemotherapy agents such as 5-fluorouracil and docetaxel has been observed to cause disruption in lacrimal drainage and MGD due to inflammation and ductal obstruction of the meibomian gland (10). Patients with head and neck cancer (not involving the orbit) who received radiation therapy were observed to have a higher rate of meibomian gland loss compared to healthy, age-matched control patients (15). MGD has also been reported in patients receiving cetuximab for colorectal cancer and bortezomib for multiple myeloma (16, 17). In another recent case report, MGD was observed in a patient with breast cancer who received HER-2-directed therapy (both trastuzumab and pertuzumab) and anastrozole (18).

The primary mechanism of MGD involves hyperkeratinization of the meibomian gland which leads to gland obstruction, reduced lipid-rich meibum output, and ultimately a tear film that is unstable and more prone to evaporation causing dry eyes (11, 19). While the etiology of the keratinization process in MGD is not fully understood, reactive oxygen species and overexpression of heat shock protein HSP90-alpha and peroxiredoxins appear to be involved (19). MGD may also be mediated by pro-inflammatory mechanisms. Increased lymphocyte density in conjunctival tissue and high proportion of CD4+ and CD8+ cells have been observed in patients with MGD (20). Analysis from tear samples of patients with MGD who were treated with intense pulse light revealed significant reduction in IL-17A and IL-6, suggesting that these cytokines may be involved inflammatory processes leading to MGD (21). MGD has also been observed in patients with Sjogren syndrome (primary and secondary) as well as in patients with ocular graft-versus-host disease following allogeneic bone marrow transplantation, highlighting the potential underlying immune-mediated mechanisms in MGD (22, 23).

In this case report, we presented a rare presentation of suspected checkpoint inhibitor toxicity: immune-mediated MGD from pembrolizumab. MGD can present with dry eyes, redness, pruritis, eye lid swelling, and other symptoms of eye irritation (11, 24). The diagnosis of MGD involves a series of examinations including tear meniscus height measurement, tear breakup time, Schirmer test, and additional tests such as corneal staining, meibomian gland expression, and meibography (11, 24). Meibomian gland expressing turbid or thick secretions is characteristic of MGD (24). Presence of atrophic meibomian glands on meibography is also a distinct finding in MGD (11). The etiology of our patient’s MGD was thought to be possibly immune-mediated due to pembrolizumab. As with other ocular irAEs, tissue biopsy was not required in this case to establish the diagnosis of immune-mediated MGD as the diagnosis was made clinically based on the timing of symptoms in relation to immunotherapy administration. Ocular irAEs can appear within weeks to months after initiating immunotherapy (1). Our patient completed eight cycles of pembrolizumab over a 5-month period, which was discontinued due to grade 3 immune-mediated pneumonitis. Her presenting ocular symptoms of eye redness developed approximately 3 months after stopping pembrolizumab. Most patients with MGD can be entirely asymptomatic (25). It is possible that our patient had asymptomatic MGD earlier on while receiving immunotherapy which clinically manifested a short period later.

To our knowledge, we present the first reported case of suspected immune-mediated meibomian gland dysfunction following immunotherapy. Brouwer et al. previously described the case of a 68-year-old man with metastatic melanoma who reported symptoms of dry eyes after four months of treatment with ipilimumab and nivolumab (26). The patient also developed grade 3 and 4 irAEs for which immunotherapy was discontinued. However, he continued to have persistent ocular symptoms 3 months after discontinuation of therapy and despite the use of artificial tears and systemic steroids that were originally prescribed for immune-mediated adrenal insufficiency. The patient was eventually seen by ophthalmology where he was found to have immune-mediated ocular rosacea that was treated with topical steroids and topical tetracycline antibiotics which improved his symptoms. His eye exam also noted meibomian gland *congestion* that was likely secondary to the ocular rosacea and resolved after three months of ocular rosacea treatment. In contrast, our patient had persistent meibomian gland atrophy with impaired function. Furthermore, our patient’s MGD was more likely due to immunotherapy given temporal relationship with immunotherapy and not secondary to an underlying ocular condition. MGD has also been reported in patients who received systemic therapy as previously mentioned. Thus, additional factors with our patient such as her previous neoadjuvant ddMVAC therapy and the non-specific nature of her underlying malignancy could have also contributed to the development of MGD. Awareness of MGD as a potential association with immunotherapy may lead to additional reports which would further establish the possibility of MGD as an irAE.

Treatment of MGD depends on the severity of symptoms. The IWMGD has proposed a staging system to classify MGD severity based on the degree of abnormal meibomian gland expression and quality of meibum, severity of symptoms, and amount of corneal staining (27). For mild cases of MGD, treatment includes eyelid warming, optimizing ambient humidity, lifestyle modifications, increased dietary omega-3 fatty acid intake, and lipid-based eye drops (27, 28). Oral tetracyclines and ophthalmic anti-inflammatories can be used in moderate to severe cases (27). Lipid-based eye drops can improve symptoms by promoting tear film stability and minimizing tear evaporation (28). Omega fatty acid supplementation is thought to enhance the lipid-rich contents of the meibum (28). Newly developed therapies for MGD such as thermal LipiFlow and intense pulsed light are also available (29). MGD is defined as a chronic condition, and permanent meibomian gland atrophy and posterior lid margin scarring can occur in severe cases (11, 29). It is possible that our patient’s MGD occurred despite systemic corticosteroid use for the preceding immune-mediated pneumonitis episode due to irreversible keratinization of the meibomian gland ducts.

## Conclusion

MGD is a potentially rare ocular immune-related adverse event from immune checkpoint inhibitor therapy. Patients can present with dry eyes, redness, and other symptoms of eye irritation similar to other ocular irAEs. The diagnosis of MGD can be supported with abnormal meibomian expression of viscous and turbid fluid as well as presence of atrophic meibomian glands on infrared meibography. Management strategies for MGD are aimed at stabilizing the tear film and minimizing evaporation with conservative therapies including eyelid warming, lipid-based eye drops, and omega fatty acid supplementation.

## Data availability statement

The original contributions presented in the study are included in the article/supplementary material, further inquiries can be directed to the corresponding author.

## Ethics statement

Ethical review and approval were not required for the study on human participants in accordance with the local legislation and institutional requirements. The patients/participants provided their written informed consent to participate in this study.

## Author contributions

CN wrote the manuscript. CS acquired/formatted the images used in the figures. CS, MM, and AA revised and gave final approval of the manuscript for submission. All authors contributed to the article and approved the submitted version.

## Acknowledgments

The authors would like to thank Dr. Ellery Isaac, OD (Birmingham Vision Care, Bloomfield Hills, MI) for providing the ocular images used in this publication.

## Conflict of interest

AA has served in an advisory role to Merck, Pfizer, AstraZeneca, and Bristol-Myers Squibb; received research funding from Genentech, Bristol-Myers Squibb, Merck Sharp & Dohme, Prometheus Laboratories, Mirati Therapeutics, AstraZeneca, Roche, Bayer, Progenics, Astellas Pharma, Arcus Biosciences, Celgene, Janssen; received travel, accommodations, expenses from Merck and Bristol-Myers Squibb.

The remaining authors declare that the research was conducted in the absence of any commercial or financial relationships that could be construed as a potential conflict of interest.

## Publisher’s note

All claims expressed in this article are solely those of the authors and do not necessarily represent those of their affiliated organizations, or those of the publisher, the editors and the reviewers. Any product that may be evaluated in this article, or claim that may be made by its manufacturer, is not guaranteed or endorsed by the publisher.
